# Antennas and Propagation: A Sensor Approach

**DOI:** 10.3390/s21144920

**Published:** 2021-07-20

**Authors:** Razvan D. Tamas

**Affiliations:** Department of Electronics and Telecommunications, Constanta Maritime University, Str. Mircea cel Batran nr. 104, 900663 Constanta, Romania; tamas@ieee.org

Antennas are essentially transducers, as they convert electromagnetic fields into signals and vice versa. Moreover, remote sensing or sensor networks cannot be imagined without antennas, and radiowave propagation in complex environments is a crucial aspect for the operation of such systems. New technologies such as 5G generate further perspectives for sensor networks and raise additional challenges to antenna design. 

This Special Issue gathers topics of utmost interest in the field of antennas and propagation, such as:

- New directions and challenges in antenna design and propagation;
- Innovative antenna technologies for space applications;
- Metamaterial, metasurface and other periodic structures;
- Antennas for 5G;
- Electromagnetic field measurements and remote sensing applications.

We originally invited the authors who contributed to the 2020 International Workshop on Antenna Technology, held in Bucharest (Romania) on 25–28 March, to submit thoroughly extended versions of their work. Surprisingly, half of the submissions to the Special Issue through the deadline were not conference paper extensions but completely new contributions.

## 1. Summary of the Special Issue

### 1.1. New Directions and Challenges in Antenna Design and Propagation

A slot-fed terahertz dielectric resonator antenna driven by an optimized photomixer is proposed in [1], and the interaction of the laser and photomixer is also studied. It is demonstrated that, in a continuous wave terahertz photomixing scheme, the generated THz power is proportional to the fourth power of the surface electric field on the photoconductive layer. The total efficiency was considerably improved due to enhancements in the laser-to-THz conversion as well as the radiation and matching efficiencies.

In Ref. [2], radio transmission and impedance matching in medical telemetry are investigated. Impedance matching inside a human body is studied both for electric and magnetic dipoles. The authors demonstrate that the implantation of a magnetic dipole is more beneficial than that of an electric dipole, as it provides impedance characteristics that are more appropriate to the human body as an antenna environment.

The simultaneous influence of the substrate anisotropy and substrate bending are numerically and experimentally investigated in [3] for planar resonators on flexible textile and polymer substrates. The effects are studied on various resonant structures with different types of slots and defected ground as well as on fractal resonators. 

In Ref. [4], the authors present small, flexible, low-profile and light-weight all-textile antennas for wearable wireless sensor networks (W-WSNs) and investigate the impact of the textile materials on the antenna performance. A step-by-step procedure for design, fabrication and measurement of small wearable backed antennas for W-WSNs is also suggested. 

The work in [5] proposes a random spreading of the scrambled timestamp sequence (STS) in ultra-wide-band (UWB) pulse communications. The timestamp estimation is improved by reducing the side lobes of the correlation. A transmission in a UWB channel with frequency selective fading shows low timestamp detection errors.

A modified, compact dipole antenna for energy harvesting applications is proposed in [6]. The design is based on a folded, short-circuited dipole radiator, and three feeding techniques are investigated. Such antenna configurations show good conversion efficiency when powering Bluetooth low-energy wireless sensors. 

### 1.2. Innovative Antenna Technologies for Space Applications

In Ref. [7], multibeam antenna systems are proposed with the aim to provide multispot coverage for broadband satellite communications in the Ka-band. Two multibeam reflectarrays are used, making it possible to halve the number of required antennas onboard the satellite. 

The work in [8] presents an antenna design that can be used as an array element for monitoring and detecting radio emissions resulting from cosmic particle interactions in the atmosphere. The proposed antenna provides a high gain over a large relative bandwidth, a narrow beamwidth, a small group delay variation and a very stable radiation pattern between 110 and 190 MHz. 

### 1.3. Metamaterial, Metasurface and Other Periodic Structures

In [9], a new design method for a planar and compact dual-band dipole antenna is proposed. The antenna has a hybrid CRLH (composite right- and left-handed) structure with lumped elements for a dual-band operation. A design for 2.4 and 5.2 GHz mobile applications is presented.

A printed edge-fed counterpart of the Bruce wire antenna array for frequency scanning applications is presented in [10]. The unit cell of the proposed antenna consists of bowtie and semi-circular elements that cover a bandwidth between 22 GHz and 38 GHz. 

The work presented in [11] focuses on design issues in the implementation of holey glide-symmetric periodic structures for waveguide-based components. An analysis of realistic hole structures is performed by using an effective hole depth method that can be used as a tool for designing electrically large waveguide-based components. 

An integration of a microstrip slot antenna array for 5.8 GHz with dye-sensitized solar cells is proposed in [12]. It is shown that the antenna array has a slight influence on the solar cell performance, and the interference of the solar cells with the antenna feeding system is also negligible. 

A new slot-based antenna system with horizontal polarization for unmanned aerial vehicle (UAV) ground control stations (GCS) is outlined in [13]. The proposed antenna system consists of coaxial cylinders and slots. The vertical slots are periodically placed around the outer cylinder and generate in-phase, horizontally polarized beams, resulting in an omnidirectional radiation pattern.

### 1.4. Antennas for 5G

In Ref. [14], a multiband antenna for microwave and millimeter-wave applications is presented. The proposed antenna consists of a slotted, conical patch connected to a small triangular patch. It can be used for wireless local area network (WLAN) applications and for fifth-generation (5G) communication devices.

In Ref. [15], the authors present a simple, compact and low-cost design method for low-profile, multi-band antennas. Such antennas can be employed in overcrowded, future generation networks in the K/Ka band. The proposed antenna structures consist of several monopoles, one for each operating frequency, along with a frequency selective feeding network. This concept leads to scalable structures suitable for 5G applications. 

In Ref. [16], a small antenna for sub-6GHz 5G communications is proposed. The design consists of a wide-band antenna connected to a small multiplexer comprising three metamaterial channel filters. It can be used on channels within three frequency bands; channel selection is experimentally demonstrated so as to prove the validity of the presented design. 

### 1.5. Electromagnetic Field Measurements and Remote Sensing Applications

In Ref. [17], the authors propose an ultra-wide band (UWB) antenna system and a direction-finding (DF) approach based on using energy-based descriptors instead of classical frequency domain parameters. The method can be applied for locating electric discharges in high-voltage power distribution systems through their electromagnetic signature in the radio frequency range.

A calibration method for high-resolution, hybrid MIMO turntable radars is presented in [18]. A line of small metal spheres is employed as a test pattern in the calibration process in order to measure the position shift caused by undesired antenna effects. The unwanted effects resulting from the near-field antenna response are analyzed, modelled and significantly mitigated, by exploiting the symmetry and response of the MIMO configuration. 

In Ref. [19], the authors show that the distance averaging technique can be applied to reduce the effect of the common mode currents for measuring the field radiated by symmetrical antennas. Two measuring configurations are proposed, depending on the number of symmetry degrees of the antenna under test. A differential approach for extracting the field created by the common mode currents is also developed. 

In Ref. [20], the authors elaborate on how a sensor using modified split ring resonators (SRRs) can be designed, simulated, fabricated and used for advanced investigation and accurate measurements of the complex permittivity of solid dielectrics. 

The work in [21] proposes a near-field to far-field transformation algorithm based on spherical wave expansion for near-field RCS measurements. Each weight in this expansion is calculated by using an iterative least squares QR factorization method. The proposed NFFFT is verified for several types of scatterers.

## Data Availability

Not applicable.
